# *In vivo* Evaluation of Nanostructured Fibrin-Agarose Hydrogels With Mesenchymal Stem Cells for Peripheral Nerve Repair

**DOI:** 10.3389/fncel.2018.00501

**Published:** 2018-12-18

**Authors:** Jesús Chato-Astrain, Fernando Campos, Olga Roda, Esther Miralles, Daniel Durand-Herrera, José Antonio Sáez-Moreno, Salomé García-García, Miguel Alaminos, Antonio Campos, Víctor Carriel

**Affiliations:** ^1^Department of Histology, Tissue Engineering Group, Faculty of Medicine, University of Granada, Granada, Spain; ^2^Doctoral Program in Biomedicine, Faculty of Medicine, University of Granada, Granada, Spain; ^3^Instituto de Investigación Biosanitaria Ibs. GRANADA, Granada, Spain; ^4^Department of Anatomy and Embryology, Faculty of Medicine, University of Granada, Granada, Spain; ^5^Division of Clinical Neurophysiology, University Hospital San Cecilio, Granada, Spain

**Keywords:** peripheral nerve repair, neural tissue engineering, fibrin-agarose hydrogels, *in vivo*, histology, mesenchymal stem cells

## Abstract

The regenerative capability of peripheral nerves is very limited, and several strategies have been proposed to increase nerve regeneration. In the present work, we have analyzed the *in vivo* usefulness of a novel nanostructured fibrin-agarose bio-artificial nerve substitute (Nano) used alone or in combination with NeuraGen^®^ collagen type I conduits (Coll-Nano) in laboratory rats with a 10-mm sciatic nerve defect. Control animals were subjected to the gold-standard autograft technique (Auto). Results first demonstrated that the percentage of self-amputations was lower in Nano and Coll-Nano groups as compared to the Auto group. Neurotrophic ulcers were more abundant in the Auto group (60%, with 66.6% of them being >2-mm) than Nano and Coll-Nano groups (0%) at 4 weeks, although Nano showed more ulcers after 12 weeks. Foot length was significantly altered in Auto animals due to neurogenic retraction, but not in Nano and Coll-Nano groups after 12 weeks. At the functional level, all animals showed a partial sensory recovery as determined by the pinch test, especially in Nano and Auto groups, but did not reach the levels of native animals. Toe-spread test revealed a partial motor function recovery only in Nano animals at 4 weeks and Auto and Nano at 12 weeks. Electromyography showed clear denervation signs in all experimental groups, with few differences between Auto and Nano animals. After 12 weeks, an important denervation decrease and an increase of the reinnervation process was found in Auto and Nano groups, with no differences between these groups. Histological analyses demonstrated an active peripheral nerve regeneration process with newly formed peripheral nerve fascicles showing S-100, GAP-43 and myelin in all experimental groups. The peripheral nerve regeneration process was more abundant in Auto group, followed by Nano group, and both were better than Coll-Nano group. Muscle histology confirmed the electromyography results and showed some atrophy and fibrosis signs and an important weight and volume loss in all groups, especially in the Coll-Nano group (56.8% weight and 60.4% volume loss). All these results suggest that the novel Nano substitutes used in *in vivo* were able to contribute to bridge a 10-mm peripheral nerve defect in rats.

## Introduction

Peripheral nerves (PNs) are delicate organs which form a highly complex network throughout the body connecting the central nervous system with distal target organs (Topp and Boyd, 2006; Carriel et al., 2014a). Histologically, PNs are composed by the nerve tissue or parenchyma and three specialized connective tissue layers or stroma (Geuna et al., 2009; Carriel et al., 2014a). The parenchyma is organized in conductive units called peripheral nerve fibers (PNFs) internally formed by neuronal axons surrounded by Schwann cells (SCs) and a thin external basal lamina. PNFs can be myelinated (in which SCs form a lipid-rich multilayered myelin sheath) or unmyelinated (Carriel et al., 2017a). About the stroma, PNs are externally covered by a collagen-rich and vascularized connective tissue layer, the epineurium. Internally, the parenchyma is organized forming fascicles which are surrounded by the perineurium. Finally, at the intrafascicular level, each PNF is surrounded by a loose connective tissue, the endoneurium (Topp and Boyd, 2006; Geuna et al., 2009; Carriel et al., 2014a, 2017a).

The structure and function of PNs can be affected by several pathological conditions, neoplasm and traumatic injuries (Dahlin, 2008; Moore et al., 2009; Carriel et al., 2014a). Following structural damage, PNs have a limited capability to regenerate their components to reestablish the motor, sensory and vegetative functions. Neoplasm removal and traumatic injuries could severely affect the PNs structure and function with a negative impact in the quality of life of patients worldwide. Incomplete or complete transections of PNs, without loss of substance, are directly repaired by neurorrhaphy in order to re-establish the nerve trunk and fascicles continuity with acceptable functional recovery (Dahlin, 2008; Carriel et al., 2014a). To repair nerve injuries with loss of substance, the use of nerve grafts (autograft or allograft) is needed (Campbell, 2008; Dahlin, 2008; Kehoe et al., 2012; Carriel et al., 2014a,b). Currently, the nerve autograft is the gold standard treatment to repair critical nerve gaps (>3-cm of length). This method provides an adequate ECM, functional SCs and growth factors which promote an efficient PN regeneration in approximately 50% of these cases (Pabari et al., 2010; Daly W. et al., 2012; Carriel et al., 2014a). However, the nerve autograft and especially the nerve allograft, have several well-known disadvantages (e.g., donor site morbidity, lack of graft material, possibility of painful neuroma, scarring, sensory loss, etc.) and their use should be limited to repair nerve gaps of approximately 5-cm length (Kehoe et al., 2012; Carriel et al., 2014a).

In this context, the tubulization technique emerged as a potential alternative for PN repair. The first generations of hollow nerve conduits, composed by natural or synthetic biomaterials, showed promising experimental results (Daly W. et al., 2012; Kehoe et al., 2012; Carriel et al., 2014a). However, once they started to be used to treat critical nerve gaps in human, failure results started to be published (Moore et al., 2009; Liodaki et al., 2013; Carriel et al., 2014a). Tubulization failure can be related to several factors, but in critical nerve defects, a reduction of growth factors diffusion occurs with the consequent failure of the regeneration process (Webber and Zochodne, 2010; Carriel et al., 2014a). Currently, tubulization is considered a safe treatment in the repair of non-critical (<3-cm) sensory nerve gaps (Moore et al., 2009; Wangensteen and Kalliainen, 2010; Boeckstyns et al., 2013; Carriel et al., 2014a). Due to the limitations and unsatisfactory results obtained with the nerve grafts and conduits, current research is focused on the generation of novel tissue engineering (TE) strategies for PN repair.

Tissue engineering combines cells with biomaterials and specific growth factors to elaborate tissue-like substitutes for the replacement or repair of damaged tissues or organs (Sanchez-Quevedo et al., 2007; Carriel et al., 2014a). In the case of the peripheral nerve TE (PNTE), the aim is to develop biological substitutes to promote and/or accelerate the intrinsic regeneration capability of damaged PNs (Geuna et al., 2010; Daly W. et al., 2012; Carriel et al., 2014a). Over the recent years, a wide range of promising TE strategies have been described (see reviews, Daly W. et al., 2012; Carriel et al., 2014a; Wieringa et al., 2018). From a physical and structural point of view, important advances were obtained by the incorporation of aligned biomaterials or nanofibers to the regenerative microenvironment (Gu et al., 2014; Pedrosa et al., 2017). From the biological perspective, authors demonstrated a significant increase of PNs regeneration through the use of functionalized and biologically active biomaterials, the incorporation of growth factors and gene-based therapies and, especially, by the incorporation of biomaterials containing different cells sources (Zheng and Cui, 2010; Ladak et al., 2011; Lopatina et al., 2011; McGrath et al., 2012; Carriel et al., 2013, 2017c; Georgiou et al., 2015). These advances suggest that the closer we get to the biomimetic regenerative microenvironment and structure, the better results we obtain (Carriel et al., 2014a; Wieringa et al., 2018).

In the field of TE, our research group developed a natural and biodegradable hybrid hydrogel composed by human fibrin and agarose type VII (Alaminos et al., 2006). This fibrin-agarose hydrogel (FAH) was successfully used to develop bioengineered cornea (Alaminos et al., 2006), oral mucosa (Sanchez-Quevedo et al., 2007), skin (Carriel et al., 2012), cartilage (Garcia-Martinez et al., 2017), PNs substitutes (Carriel et al., 2013, 2017c) and other tissue-like structures (Campos et al., 2016, 2017). Regarding the use of FAH in PNTE, these biomaterials were used alone and in combination with adipose-derived mesenchymal stem cells (ADMSCs) as intraluminal fillers of biodegradable NeuraGen^®^ conduits to repair 10-mm nerve gap in rats (Carriel et al., 2013, 2017c). These studies demonstrated that the incorporation of acellular FAH hydrogels and, especially, FAH containing ADMSCs, provided a suitable regenerative microenvironment which resulted in a significant improvement of the clinical, functional, electromyographic and histological profiles (Carriel et al., 2013, 2017c). These studies suggest that NeuraGen^®^ conduits and cellular FAH contributed synergistically to the PN regeneration process and reinnervation of distal target organs. Despite these positive results, FAH does not have the adequate biomechanical properties to be directly used in PN reconstruction. In this context, it was recently demonstrated that the biomechanical and structural properties of FAH can be significantly improved in a controlled manner with the nanostructuration technique (Campos et al., 2016, 2017). This methodology promotes nanoscale molecular interactions among the biomaterial fibers, and it was recently applied to generate novel nanostructured fibrin-agarose bio-artificial nerve substitutes (NFABNS) (Carriel et al., 2017d). This fabrication process allowed to successfully recreate the size and shape of PNs with promising structural and biomechanical properties (Carriel et al., 2017d). In addition, *ex vivo* characterization demonstrated that NFABNS were cytocompatible, supporting human ADMSCs viability, proliferation and function over the time (Carriel et al., 2017d). However, the potential clinical usefulness of NFABNS has not been studied yet.

Due to the promising structural and biological properties offered by novel NFABNS for PN reconstruction, and in view of the putative synergetic effects of FAH and NeuraGen^®^ collagen conduits, we have carried out an *in vivo* study to determine the usefulness of these devices in PN repair. For these reasons, the aim of this study was to evaluate the possibility to bridge 10-mm nerve gaps in rats by using NFABNS and NFABNS as intraluminal fillers of NeuraGen^®^ conduits. Furthermore, the PN regeneration profile was determined through the use of clinical, functional, electromyography, histological, and ultrastructural studies.

## Materials and Methods

### Laboratory Animals

In this study, 20 male 13-week-old Wistar rats weighing 250–300 g (at the beginning) were obtained from JANVIER LABS^®^ and kept under veterinary and technical supervision in the animal facility of the University Hospital *Virgen de las Nieves, Granada, Spain*. Animals were housed in a light and temperature-controlled room (∼21°C and 12 h light/dark) with *ad libitum* access to standard rat chow and tap water. In this study, 15 animals were subjected to surgical procedures using general anesthesia by intraperitoneal injection of a mixture of acepromazine -Calmo-Neosan^®^ 0.001 mg per g of weight of the animal-, ketamine -Imalgene 1000^®^ 0.15 mg per g of weight- and atropine -0.05 μg of per g of weight-. At the beginning of the study, we harvested a small biopsy (∼1-cm^3^) from the inguinal pad fat for isolation of autologous ADMSCs. Once enough amounts of ADMSCs were obtained, PNs substitutes were generated. In addition, five healthy animals were used as control native (CTR-Native). At the end of this study, all animals were euthanatized by overdose of anesthesia. All procedures were performed according to the European Union and Spanish Government guidelines for the ethical care of animals (EU Directive No. 63/2010, RD 53/2013) and the research projects were approved by the ethical and experimentation committee of Granada (FIS PI14/01343 and FIS PI17/0393).

### Adipose-Derived Mesenchymal Stem Cells Isolation and Culture

The autologous ADMSCs were isolated and cultured following previously described protocols (Carriel et al., 2013, 2017d). Concisely, adipose tissue biopsies were mechanically fragmented into small pieces and digested with 0.3% type I collagenase solution (Gibco BRL Life Technologies) for 8 h at 37°C. Once the ECM was digested, the cells were isolated by centrifugation and then cultured in basal medium [Dulbecco’s modified Eagle’s medium (DMEM)] supplemented with 10% fetal bovine serum (FBS; Sigma-Aldrich) and antibiotic-antimycotics cocktail solution (100 U/ml penicillin G, 100 μg/ml streptomycin, and 0.25 μg/ml amphotericin B, Sigma-Aldrich, Steinheim, Germany). Cells were kept under standard culture conditions (37°C and 5% CO_2_), the culture media was renewed every 3 days and cells expanded until passage 4.

**FIGURE 1:**
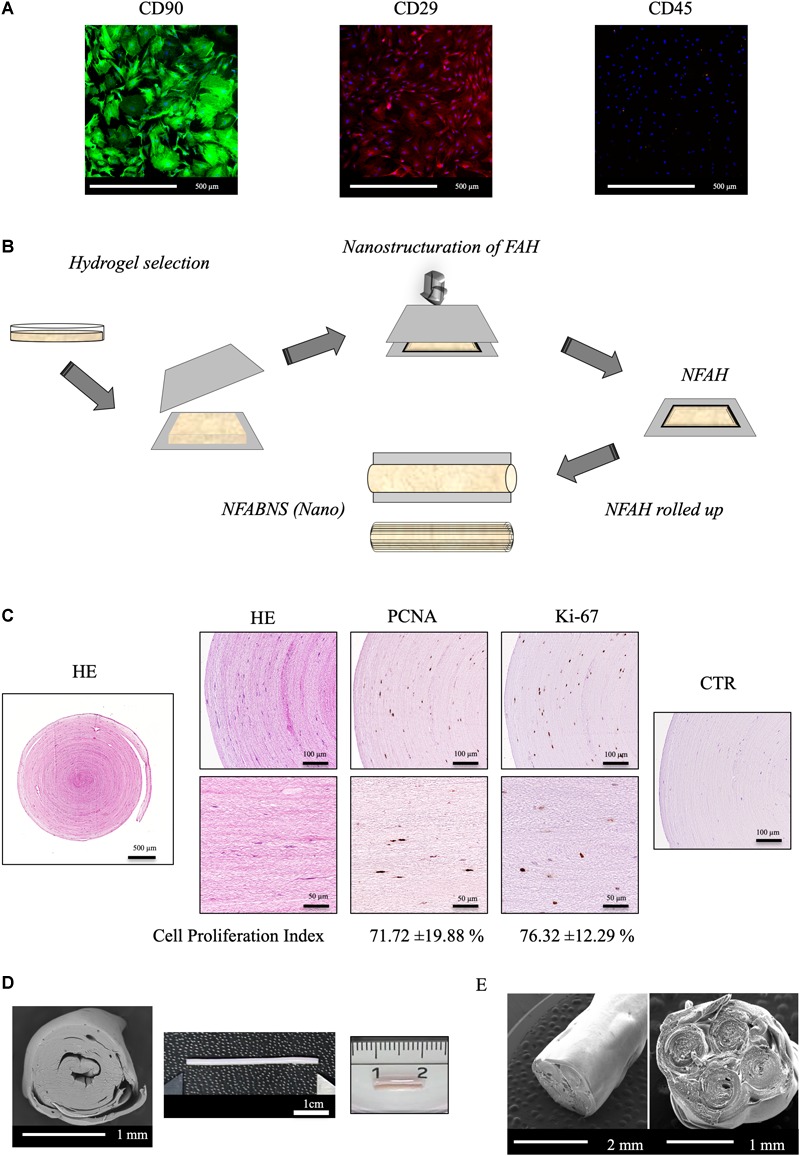
ADMSCs characterization and NFABNS generation. **(A)** Shows representative images of the immunostaining pattern of stemness markers of the rat ADMSCs. **(B)** Shows how from a square uncompressed FAH it is possible to fabricate a cylindrical multilayered NFABNS of desire dimensions. **(C)** Exhibit the histological pattern of NFABNS, ADMSCs distribution with HE and the presence and percentages of proliferating cells (cell proliferation index) detected by immunohistochemistry for PCNA and Ki-67 markers, respectively. **(D)** Shows the macroscopic and scanning electron microscopy aspect of the NFABNS used to repair the nerve defect in this study. In **(E)** scanning electron microscopy images show examples of multifasciculated NFABNS that can be generated with this methodology if needed.

The stemness profile of the rat ADMSCs used was determined by flow cytometry and immunofluorescence as previously reported (Sun et al., 2011; Lotfy et al., 2014). Flow cytometry was performed using a NovoCyte^®^ 1000 Flow Cytometer (ACEA, Biosciences Inc., United States) and cells demonstrated to be positive for CD 90 (99.43%) and CD 29 (99.67%) and negative for CD 45 (0.07%) markers. Immunofluorescence representative images and the technical information of the antibodies used can be found in Figure 1 and Table 1, respectively.

**Table 1:** Antibodies used for flow cytometry and immunostaining.

Antibody	Dilution	Pretreatment	Application	Cat. No.
FITC/Mouse monoclonal anti-CD90	1:300	–	FC	BioLegend, San Diego, CA, United States
(Clone OX-7)	1:200		IF	cat. no. 202503
PerCP/Cy5.5/Hamster	1:75	–	FC	BioLegend, San Diego, CA, United States
anti-CD-29	1:50		IF	cat. no. 102227
PE/Mouse monoclonal anti-CD-45	1:100	–	FC/IF	BioLegend, San Diego, CA, United States cat. no. 202207
Mouse monoclonal anti-PCNA (clone PC10)	1:1000	Citrate buffer, pH 6, 95°C for 25 min	IHC	Sigma-Aldrich, Steinheim, Germany cat. no. P8825
Rabbit monoclonal anti-Ki67	–	EDTA buffer pH 9, 95°C for 25 min	IHC	Master Diagnostica, Granada, Spain cat. no. MAD-000310QD
Rabbit polyclonal anti-S100	1:400	Citrate buffer, pH 6, 95°C for 25 min	IHC	Dako Cytomation, Glostrup, Denmark cat. no. Z0311
Rabbit polyclonal anti-GAP-43 (ab-41)	1:50	EDTA buffer, pH 9, 95°C for 25 min	IHC	Sigma-Aldrich, Steinheim, Germany cat. no. Sab4300525
Horse anti-mouse/rabbit IgG (peroxidase)	–	–	IHC	Vector Laboratories, CA, United States cat. no. MP-7500

*FC, flow cytometry; IF, immunofluorescence; IHC, immunohistochemistry.*

### Fabrication of NFABNS

First, FAHs containing ADMSCs were elaborated following previously described protocols (Carriel et al., 2013, 2017d). Briefly, to prepare 10 ml of FAH a mixture composed by 7.6 ml human plasma, 0.15 ml tranexamic acid (Amchafibrin, Fides-Ecofarma, Valencia, Spain) and 1.25 ml of basal medium containing ADMSCs (5 × 10^4^ cells/ml) was prepared. This solution was mixed and 1 ml of 2% CaCl_2_ and 0.5 ml of melted 2% type VII agarose were added, carefully mixed, placed in 60-mm petri dishes and kept under standard culture condition until complete gelation (∼1 h). This procedure resulted in the generation of 10 ml of uniform FAH containing ADMSCs with specific dimensions (5-mm of thickness and 6-mm of diameter). After gelation, 5 ml of basal medium was added to each construct and they were kept in culture for 24 h and then used for the fabrication of NFABNS.

To generate the NFABNS, we followed a well-described protocol, which allowed us to produce customized substitutes with specific biomechanical properties and dimensions ensuring the cellular viability and functionality (Carriel et al., 2017d). Briefly, to fabricate these NFABNS, FAHs were carefully harvested from the petri dishes and then nanostructured. Hydrogels were cut symmetrically (3-cm × 3-cm × 3-cm) and then placed between a couple of nylon filters membranes (0.22 μm) and Whatman 3-mm absorbent papers below a flat glass surface. Immediately, a uniform and homogeneously distributed mechanical pressure (500 g) was applied for 2.5 min obtaining a highly dense nanostructured FAH (NFAH) of 50–60 μm thickness (Scionti et al., 2014; Carriel et al., 2017d) (Figure 1). At this point, it was possible to fabricate NFABNS with specific dimensions (length and/or diameter) or number of fascicles (uni-fascicular or multi-fascicular) (*see examples of each in* Figure 1). Here, unifascicular NFABNS composed by multilayered NFAH of 1-cm long and ∼1.5-mm or 1-mm diameter were generated based on the dimensions of the adult rat sciatic nerve, and according to the length of the nerve gap created (10-mm).

This methodology allowed us to generate NFABNS with 0.30 ± 0.04 MPa of Young’s Modulus, 0.42 ± 0.03 MPa of stress at fracture and 169.6 ± 9.85% strain at fracture (deformation) mean values, as previously characterized (Carriel et al., 2017d). Furthermore, to ensure the viability of the ADMSCs contained in the NFABNS, cell proliferation was determined by immunohistochemistry for PCNA and Ki-67 after 48 h of culture (Figure 1) as described previously (Carriel et al., 2017d). In this sense, the cell proliferation index of the ADMSCs was 71.72% for PCNA and 76.32% for Ki-67. The technical information of the antibodies used is summarized in the Table 1.

### Surgical Procedures and Experimental Study Groups

Initially, 15 animals were subjected to general anesthesia (as described above) and then a segment of 10-mm was removed from the left sciatic nerve. The right hind leg was used as non-operated control in all cases (CTR). Animals were then randomly assigned to the following experimental groups (*n* = 5 in each):

– Autograft control group (Auto), where the removed fragment of the nerve was rotated 180° and used to bridge the nerve gap.
– Nano group (Nano), in which the nerve gap was microsurgically bridged by using 10-mm NFABNS of ∼1.5-mm diameter.
– Collagen Nano group (Coll-Nano), where the nerve defects were repaired with NeuraGen^®^ collagen type I conduits (Integra^®^ Life Sciences Corp., Plainsboro, NJ, United States) filled with an NFABNS of 10-mm of ∼1-mm diameter.
– Control native (CTR-Native), where healthy animals were used for comparisons.

For all surgical procedures 7/0 Prolene (polypropylene, blue monofilament) suture material was used. After the nerve microsurgical repair and wound closure, all animals were housed as mentioned above and each one received analgesic treatment (metamizole) in the drinking water for 48 h. In this study, animals were subjected to a two-time clinical assessment and electromyography (EMG) at 4 and 12 weeks after surgery, respectively. The analyses at 4 weeks were performed to confirm the clinical and functional impact of nerve injury, whereas the aim of the 12 weeks evaluation was to accurately determine differences among groups during an active regeneration and partial functional recovery, as recommended in the literature (Geuna, 2015). Following the second EMG, animals were housed for other 14 days and then euthanized. This period was used to favor muscle healing (hemorrhage and inflammation due to the EMG) for further morphometric and histological analyses. Therefore, all histological analyses were performed at 14 weeks after surgery.

The NeuraGen^®^ conduits were chosen due to their well-known positive impact on PNs regeneration when combined with FAH and ADMSCs (Carriel et al., 2013, 2017c). Furthermore, these conduits are FDA approved and they are currently one of the most frequently used nerve guides in the clinical practice (Wangensteen and Kalliainen, 2010; Krarup et al., 2017).

### Clinical Assessment

In order to determine the sensory and motor function profile after PN repair, animals were subjected to a series of well-known tests at 4 and 12 weeks after surgery (Vleggeert-Lankamp, 2007; Siemionow et al., 2011; Carriel et al., 2013). In this regard, the following analyses were performed: evaluation of toes self-amputations in the operated leg; the percentage and size of plantar neurotrophic ulcers (≤2-mm/>2-mm); the foot length (in mm), as indicator of the neurogenic retraction of the muscles innervated by the sciatic nerve; the pinch test of sensory recovery; and the toe-spread test.

For the pinch test, a mild pinching stimulus was applied to the skin of the operated leg from the toe to the knee joint, until a withdrawal reaction was observed. This reaction was graded on a four-point scale as follows: 0 = no withdrawal response, 1 = response to stimulus above the ankle, 2 = response to distal stimulation to the ankle in the heel/plantar region and 3 = response to stimulation in the metatarsal region as previously described (Siemionow et al., 2011; Carriel et al., 2013).

The toe-spread test consisted in the evaluation of the extension and abduction reaction of the toes during tail-suspension. These results were graded on a four-point scale as follows: 0 = no toe movement, 1 = some sign of toe movement, 2 = toe abduction, and 3 = toe abduction with extension (Vleggeert-Lankamp, 2007; Siemionow et al., 2011; Carriel et al., 2013).

For foot length, the rat’s hind feet were dipped in a blue ink, and the animals were permitted to walk down the walking pathway on a Plexiglas^®^ device (1-m length, 10-cm width and 15-cm height) covered with white paper, leaving its hind footprints on the paper. The foot length was measured as the distance from the heel to the third toe.

### Electromyography

All animals were subjected to EMG tests 4 and 12 weeks after the surgical procedure as previously described (Carriel et al., 2013). Briefly, animals were mildly anesthetized (1/10 of the doses used for general anesthesia) with ketamine and acepromazine to study the muscle function at rest. Furthermore, the spontaneous electrical activity of the gastrocnemius (lateral and medial) and tibialis anterior muscles was determined. These muscles were analyzed using concentric-needles and a Topas 4-channel electromyograph (Schwarzer GmbH R, Munich, Germany) with band-pass filter settings of 5–5,000 Hz. Each muscle was subjected to three measurements in three different areas. Denervation and reinnervation results were scored using a four-point scale as follows: 0 = absent (no signs in any of the three muscle areas); 1 = mild (signs in one of the three areas); 2 = moderate (signs in two areas), or 3 = severe (signs in all three areas). These analyses were carried out and interpreted by three independent experts (EM, SGG, JAS) blinded to the experimental groups. The percentage of animals with specific denervation or reinnervation degrees was calculated for each study group. The right leg of each operated animal (CTR) and both legs of independent unoperated animals (CTR-Native) were also analyzed as controls.

### Muscle Morphometric Evaluation

In order to assess the degree of atrophy of the muscles innervated by the operated left sciatic nerve, the weight (*w*) and volume (*v*) of the whole lower leg was measured at 14 weeks after surgery. Lower legs were harvested after the intracardiac perfusion (see details below). The lower legs [which contain several muscles exclusively innervated by the sciatic nerve trunk (Greene, 1968)] were exposed and then disjointed from the knee and ankle. For the *w* assessment, the dissected legs were removed from the fixative, dried in absorbent paper and weighed in a digital weighing machine (Sartorius BP 121S, precision: 0.1 mg, Sigma–Aldrich). For the *v* assessment, dissected legs were immersed in test tube (50 ml) containing 30 ml of PBS and the increase of this volume represented the lower legs volume. In this study, the percentage of *w* and *v* loss was calculated between the operated and the contralateral-side leg from each animal, including healthy animals (CTR-Native), and these values were used for statistical comparisons.

### Macroscopy, Histological, and Ultrastructural Analyses

First, animals under general anesthesia received an intraperitoneal heparin injection and then were euthanized by anesthesia overdose. After that, animals were perfused with 500 ml of saline solution followed by 500 ml of 4% neutral buffered paraformaldehyde. Perfused animals were used to evaluate the macroscopic aspect of the repaired nerves and then nerves, implants and muscle were harvested for histology.

Macroscopic analysis was aimed to evaluate nerve continuity, uniformity, adherences, or inflammatory reactions. For the histological analyses, healthy PNs and implants were carefully harvested, sectioned transversally and the central portion was obtained for light and transmission electron microscopy (TEM) analyses. For light microscopy, samples were immersed in fixative for another 24 h. In the case of muscles, following the morphometric evaluation (described above), the tibialis anterior and gastrocnemius muscles were dissected from both legs. Muscles were immersed in fixative for 24 h, sectioned transversely and then fixed for another 24 h (a total of 48 h of chemical fixation). All fixed tissues were placed in histological cassettes embedded in paraffin and sectioned at 5 μm of thickness (Carriel et al., 2017a,b).

In this study, all sections were stained with Hematoxylin and eosin (HE) for general histology. In addition, the MCOLL histochemical method was used to evaluate the general histology during the remyelination and collagen fibers reorganization processes in PNs and implants as described previously (Carriel V. et al., 2011; Carriel et al., 2013, 2014b, 2017a). The presence of SCs and newly formed axonal sprouts were evaluated by indirect immunohistochemistry for S-100 protein and GAP-43, respectively, as previously described (Carriel et al., 2013, 2014b, 2017c).

In order to determine the degree of muscle atrophy and fibrotic stromal reaction due to PN repair, the transversal sections of the tibialis anterior and gastrocnemius muscles were stained with picrosirius (PS) and Masson trichrome (MST) methods (Carriel V.S. et al., 2011; Philips et al., 2018b). Furthermore, histological sections from healthy muscles were used as native control.

For TEM analysis small tissue samples were obtained immediately after intracardiac perfusion and postfixed with 2.5% neutral buffered glutaraldehyde followed by 2% osmium tetroxide (Carriel et al., 2017d). Fixed samples were dehydrated, embedded in epoxy resin. Ultrathin sections were stained with uracil acetate and lead citrate, transferred to mesh grids and analyzed in a JEOL JEM 1200 EX II or a Carl Zeiss SMT LIBRA^®^ 120 PLUS transmission electron microscopes.

### Statistical Analyses

All quantitative data obtained from clinical assessments and muscle morphometry were analyzed using the Shapiro–Wilk test of normality. All non-normally distributed variables (toe spread, pinch test, volume loss, weight loss, electromyogram results) were compared with the Mann–Whitney non-parametric test. In the case of ulcers and amputations, variables which were expressed in percentage, the Fisher exact test was used for statistical comparisons. In all cases, *p* < 0.05 values were considered statistically significant in two-tailed tests. All data and statistical comparisons were calculated with the SPSS 16.0 software.

## Results

### Implantation and Macroscopic Postsurgical Aspect

**FIGURE 2:**
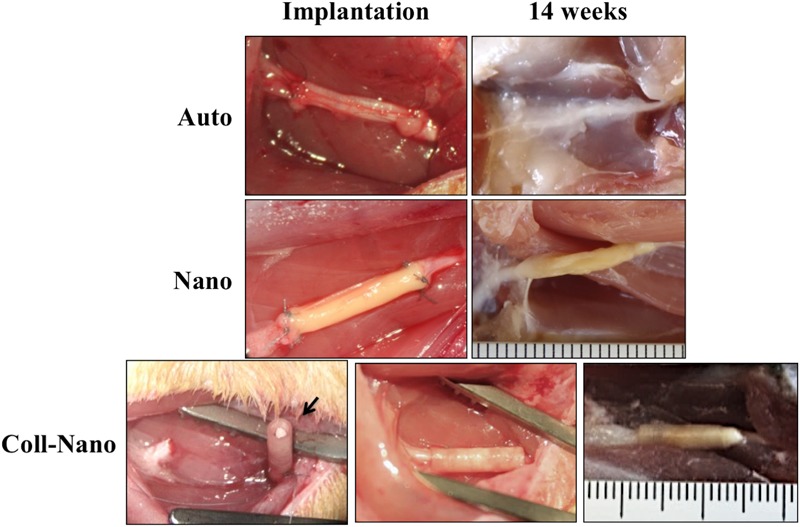
Implantation and postoperative aspect of repaired sciatic nerves. These images show the macroscopic aspect of repaired nerves and the postoperative results for autografts (Auto), NFABNS (Nano) and collagen conduits filled with one NFABNS (Coll-Nano). In all cases the nerve gap was successfully bridged after 14 weeks. In Coll-Nano group, the collagen conduits were first sutured in one of the nerve stump, filled with one 1-mm diameter NFABNS (arrow) and then the opposite nerve stump was inserted and sutured into the conduits. Please note that all images at 14 weeks were taken after perfusion.

In this study, one of the aims was to determine if, from the structural and surgical perspective, the NFABNS (Nano) and the NFABNS used as intraluminal fillers of collagen conduits (Coll-Nano) were suitable alternatives to bridge a 10-mm sciatic nerve gap in rats. The methodology used allowed to generate NFABNS with comparable size (length and diameter), shape and consistency than the target nerves. From the surgical point of view, NFABNS were easy to handle during implantation process and allowed us to repair the nerve gaps in a comparable time and technique than nerve autograft (Figure 2). Regarding the Coll-Nano group, the NFABNS were easily incorporated as intraluminal fillers of collagen conduits during surgery, and the defects were repaired by using the conventional tubulization technique without any technical inconvenience. After 14 weeks of *in vivo* implantation the Auto group showed a complete repair of the defects without any sign of structural damage, although a loose connective tissue was observed covering the repaired area (Figure 2). In the case of the Nano group, the macroscopic analysis revealed that NFABNS allowed to bridge the nerve defects successfully without signs of adherences or local adenopathies (Figure 2). Similarly, the postsurgical analysis confirmed that collagen conduits with NFABNS were able to successfully bridge the gap and the collagen conduits remained in the surface without any adherence, compression or conduit deformation (Figure 2).

### Clinical Results

In this study, clinical and functional parameters were evaluated at 4 and 12 weeks after PN repair, and results are shown in Table 2. The percentages of self-amputations at 4 weeks were higher in Auto group (80%) as compared to Nano and Coll-Nano groups (20% each). However, differences were not statistically significant between experimental groups, except for the comparison between Auto vs. CTR-native group (*p* < 0.05, Table 2). Interestingly, the percentage of self-amputations increased in the Nano and Coll-Nano groups (reaching to 60% and 40%, respectively), and differences were only significant for comparisons between CTR-Native group vs. Auto and Nano groups (*p* < 0.05, Table 2).

**Table 2:** Quantitative clinical and functional recovery results.

Groups *n* = 5 each	% Self-amputations	% Neurotrophic ulcers	% Neurotrophic ulcers (>2 mm)	Foot length (mm)	Pinch test	Toe spread test
**4 weeks postsurgical analyses**						
CTR-native	0.00	0.00	0.00	–	3.00 ± 0.00	3.00 ± 0.00
Auto	80.00^a^	60.00^a,b,d,e^	66.67^a,b,d,e^	–	1.60 ± 0.55^a,e^	0.00 ± 0.00^a^
Nano	20.00^e^	0.00^b,e^	0.00^b^	–	2.00 ± 0.71^a,c,e^	0.40 ± 0.55^a^
Coll-Nano	20.00^e^	0.00^d^	0.00^d^	–	0.80 ± 0.84^a,c^	0.00 ± 0.00^a^
**12 weeks postsurgical analyses**						
CTR-native	0.00	0.00	0.00	39.17 ± 2.04	3.00 ± 0.00	3.00 ± 0.00
Auto	80.00^a^	20.00^e^	100.00^a,b,d,e^	34.01 ± 2.47^a,d^	1.00 ± 0.71^a,e^	0.20 ± 0.45^a^
Nano	60.00^a,e^	40.00^e^	50.00^b,e^	37.39 ± 2.57	1.6 ± 0.55^a,e^	1.00 ± 0.71^a,c^
Coll-Nano	40.00^e^	0.00	0.00^d^	40.04 ± 1.67^d^	1.40 ± 0.55^a^	0.00 ± 0.00^a,c^

*The table shows all quantitative results conducted at 4 and 12 weeks followed peripheral nerve repair. Self-amputations and neurotrophic ulcers results are expressed in percentage, while the foot length, pinch and toe spread tests results are shown as mean ± standard deviation values. In this study, *p* < 0.05 values were considered statistically significant in two tailed tests. Significant differences are indicated as follows: ^*a*^Significant differences with CTR group. ^*b*^Significant differences between Auto vs. Nano group. ^*c*^Significant differences between Nano vs. Coll-Nano group. ^*d*^Significant differences between Auto vs Coll-Nano group. ^*e*^Significant differences between 4 weeks vs 12 weeks for each condition.*

Assessment of plantar neurotrophic ulcers after 4 weeks revealed that only animals from Auto groups developed these injuries (60% of animals), being these values significantly higher (*p* < 0.05) than Nano and Coll-Nano groups, where these injuries did not occur. In addition, the 66.6% of these ulcers (Auto group) were higher than 2-mm (Table 2). The analysis after 12 weeks showed a decrease of the presence of ulcers in the Auto group (from 60 to 20%) and an increase in the Nano group (from 0 to 40%), while in Coll-Nano group values were not altered (0%). However, differences among groups were not statistically significant (Table 2). Curiously, 100% of ulcers of Auto group and the 50% of Nano group were higher than 2-mm, being the differences between Auto vs. Nano and Coll-Nano groups statistically significant (*p* < 0.05, Table 2).

Evaluation of neurogenic retraction of the muscles innervated by the sciatic nerve was evaluated through the measurement of foot length at 12 weeks. This analysis revealed lower foot length values, meaning neurogenic retraction, in Auto group (34.01-mm), followed by the Nano Group (37.39-mm) and then the Coll-Nano group (40-mm). Differences were only significant for comparisons between CTR-Native group vs. Auto group and for Auto vs. Coll-Nano group (*p* < 0.05, Table 2).

The pinch test of sensory recovery after 4 weeks showed higher values, better sensory recovery, in Nano group (2/3), than the Auto (1.6/3) and Coll-Nano (0.8/3) groups. Although signs of sensory recovery were observed, especially in Nano group, these values were significantly lower (*p* < 0.05) than the sensory reaction observed in healthy animals (CTR-Native group) (3/3). In addition, when we compared these values between experimental groups differences between Auto vs. Nano group were not significant (*p* > 0.05), but differences were statistically significant when we compared Nano vs. Coll-Nano groups values (*p* < 0.05, Table 2). The pinch test of sensory recovery after 12 weeks showed a slight decreased of these values in the Auto (from 1.6/3 to 1/3) and Nano (from 2/3 to 1.6/3) groups and a slight increase in Coll-Nano group (from 0.8/3 to 1.4). Differences among these groups were not statistically significant (*p* > 0.05, Table 2), and all operated animals showed significantly lower values than the CTR-native group (*p* < 0.05, Table 2).

Finally, assessment of the motor function of digital muscle with the toe spread test revealed that all operated animals were far to be comparable to the motor response of healthy animals at 4 and 12 weeks, and differences were statistically significant (*p* < 0.05, Table 2). Interestingly, after 4 weeks slight signs of motor function were only observed in Nano group (0.4/3), but these values were not significantly higher (*p* > 0.05) than the values observed in Auto and Coll-Nano groups (Table 2). When motor function was evaluated after 12 weeks these values were increased in the Nano group (from 0.4/3 to 1/3) and slight increase in the Auto group (from 0/3 to 0.2/3), but no signs of motor function recovery were observed in Coll-Nano group (0/3), which was significantly lower than Nano group (*p* < 0.05, Table 2).

### Electromyography Results

First, the analysis of the right leg of each operated animal (CTR) and both legs of independent animals not subjected to surgery (CTR-Native group) revealed a normal recruitment pattern with normal motor unit potentials and no spontaneous activity at rest. In contrast, the EMG analysis of the experimental groups showed a wide variation of denervation and reinnervation signs (Table 3).

**Table 3:** Electromyography profile of muscles innervated by repaired sciatic nerves.

Groups *n* = 5 each	Muscles	% Denervation				% Reinnervation			
		0	1	2	3	0	1	2	3
**Electromyography after 4 weeks of peripheral nerve repair**								
Auto	Gastrocnemius lateral	40	40	20	0	0	67	33	0
	Gastrocnemius medial^c^	0	80	20	0	0	100	0	0
	Tibialis anterior^c^	0	20	80	0	0	100	0	0
Nano	Gastrocnemius lateral^a,f^	0	20	40	40	100	0	0	0
	Gastrocnemius medial^d,f^	20	0	40	40	100	0	0	0
	Tibialis anterior^d,f^	0	20	0	80	100	0	0	0
Coll-Nano	Gastrocnemius lateral^a^	0	0	20	80	100	0	0	0
	Gastrocnemius medial^a,c,d^	0	0	0	100	100	0	0	0
	Tibialis anterior^a,c,d^	0	0	0	100	100	0	0	0
**Electromyography after 12 weeks of peripheral nerve repair**								
Auto	Gastrocnemius lateral	100	0	0	0	0	60	40	0
	Gastrocnemius medial^c^	0	100	0	0	20	40	40	0
	Tibialis anterior^c^	100	0	0	0	20	60	20	0
Nano	Gastrocnemius lateral^a,b,f^	0	60	40	0	0	60	40	0
	Gastrocnemius medial^b,f^	20	40	40	0	0	80	20	0
	Tibialis anterior^a,f^	0	20	80	0	0	80	20	0
Coll-Nano	Gastrocnemius lateral^a,b,d^	0	0	60	40	60	40	0	0
	Gastrocnemius medial^a,b,c^	0	20	20	60	80	20	0	0
	Tibialis anterior^a,c^	0	20	60	20	60	40	0	0

*Results are shown as the percentage of animals of each experimental group with specific denervation or reinnervation signs in the gastrocnemius (lateral and medial) and tibialis anterior muscles at 4 and 12 weeks after surgery. Each muscle was subjected to three measurements in three different areas, and the results were scored as follows: 0 = absent (no signs in any of the three muscle areas); 1 = mild (signs in one of the three areas); 2 = moderate (signs in two areas), or 3 = severe (signs in all three areas). In this study, *p* < 0.05 values were considered statistically significant in two-tailed tests. Significant differences are indicated as follows: ^*a*^Significant differences in denervation results with Auto group for each time. ^*b*^Significant differences in denervation between Nano group vs. Coll-Nano group for each time. ^*c*^Significant differences in denervation between 4 weeks vs. 12 weeks for each group. ^*d*^Significant differences in reinnervation results with Auto group for each time. ^*e*^Significant differences in reinnervation between Nano group vs. Coll-Nano group for each time. ^*f*^Significant differences in reinnervation between 4 weeks vs. 12 weeks for each group.*

At a follow-up period of 4 weeks, most experimental groups showed clear signs of denervation (Table 3). The percentage of muscle denervation (gastrocnemius and tibialis anterior) was more severe in the Coll-Nano group (100% of tibialis anterior and gastrocnemius medial muscles were severely denervated) followed by Nano and Auto group, respectively (Table 3). Differences were statistically significant for the comparison of Coll-Nano group vs. Auto group for all analyzed muscles (*p* < 0.05) and for the gastrocnemius muscle for the comparison of Nano vs. Auto groups (*p* < 0.05). Curiously, some signs of reinnervation in the gastrocnemius and tibialis anterior muscles were observed in Auto group at this stage (Table 3).

Analysis of animals after 12 weeks after the surgical procedure revealed significant changes in the percentage of denervation and, specially, in the reinnervation profile of all experimental groups (Table 3). In the Auto group, a significant decrease of the muscle denervation percentage was observed for all muscles, and none of the animals showed denervation signs for the gastrocnemius medial and tibialis anterior muscles at this time. These values were accompanied by a slight, but not statistically significant (*p* > 0.05, Table 3), increase of the reinnervation profile in this Auto group. The EMG profile of the Nano group revealed a slight non-significant decrease of the denervation profile, with none of the muscles being severely denervated after 12 weeks. In this group of animals, we found a significant improvement (*p* < 0.05) of the reinnervation profile of all muscles at 12 weeks of follow-up, and the reinnervation profile of the gastrocnemius lateral muscle was comparable to the Auto group at this time (Table 3). Although differences were non-significant (*p* > 0.05), the reinnervation profiles of gastrocnemius medial and tibialis anterior were slightly superior in Nano group as compared to the Auto group (Table 3). Finally, the analysis of the Coll-Nano animals at 12 weeks demonstrated a slight non-significant improvement (*p* > 0.05) of the denervation profile found after 4 weeks. However, none of the muscles showed reinnervation signs and did not differ from results found at the previous stage (Table 3).

### Peripheral Nerve Regeneration Histology and Ultrastructure

**FIGURE 3:**
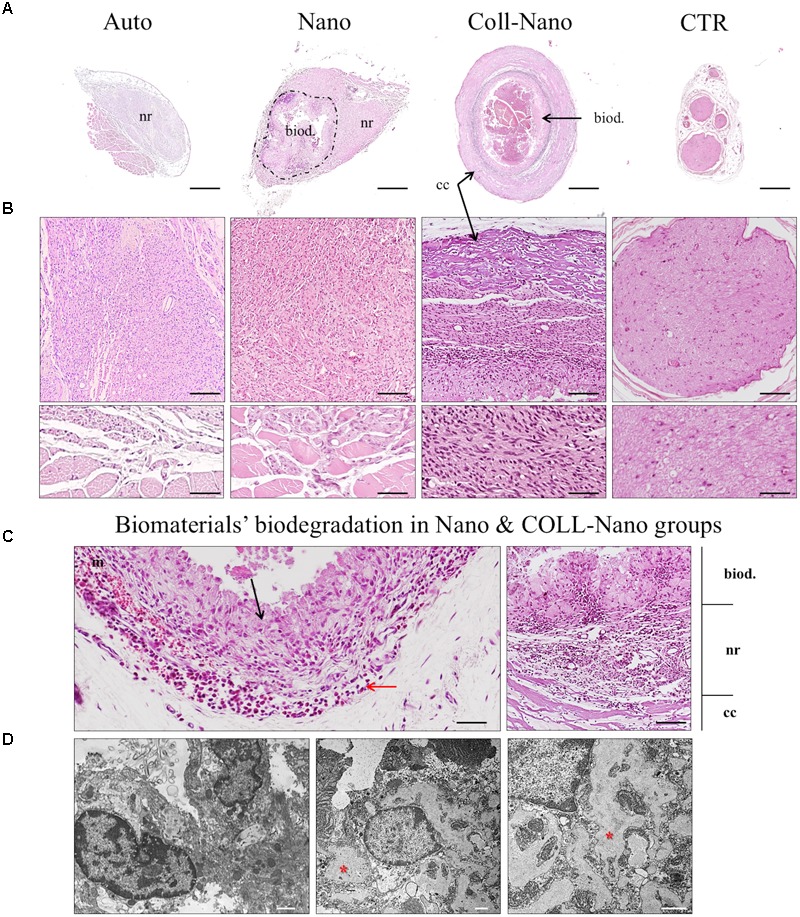
Microscopic results of peripheral nerve regeneration and biomaterials biodegradation. Figures show HE cross-section staining at low **(A)** and middle magnifications **(B,C)** from the central portion of repaired nerves by autografts (Auto), NFABNS (Nano) and collagen conduits filled with NFABNS (Coll-Nano). In addition, a representative transversal histological section of a healthy nerve (CTR) was included. In **(A)** a general overview of the regeneration process is provided, where nerve regeneration is indicated as (nr) in all groups. In Nano and Coll-Nano groups the active biodegradation of FAH is indicated with (biod.) whereas the collagen conduit in Coll-Nano with (cc). Images in **(B)** show with moderate magnification the histological pattern of peripheral nerve regeneration in each group and also the native control. In Auto and Nano groups some disperse regenerating nerve fascicles were found associated to the surrounding skeletal muscle (insets). Figures in **(C)** show representative images of the biodegradation of the FAH [in Nano (left) and Coll-Nano (right) groups] and collagen conduit (Coll-Nano group) by host macrophages (black arrows). In association to the macrophages, it was also observed a variable amount of mononuclear cells (red arrows). In right image corresponding to Coll-Nano group the three well differentiated zones are indicated, the biodegradation of FAH (biod.), peripheral nerve regeneration zone (nr) and the biodegradation of the collagen conduit (cc). TEM images in **(D)** confirmed the presence of active macrophages in Nano (left) and Coll-Nano (middle and right) groups, where large phagosomes are indicated by red asterisks. Scale bar = 500 μm in **(A)**, 100 μm and 50 μm (insets) in **(B)**, 50 μm (left) and 100 μm (right) in **(C)** and 1 μm in **(D)**.

The histological analysis carried out with HE staining at 14 weeks of the middle portion of repaired nerves confirmed an active regeneration process in all experimental groups. In the case of nerve gaps repaired with autograft technique (Auto group), the regeneration process was observed mainly at the intrafascicular level, but also in the epineural connective tissue of the graft. In this group, the process was characterized by the presence of relatively small newly formed nerve fascicles containing PNFs. Furthermore, no sign of inflammatory reaction was find in any animals (Figures 3A,B). Interestingly, it was often observed a variable number of regenerating fascicles between the surrounding rhabdomyocytes confirming some degree of dispersion of the PN regeneration process (Figure 3B, inset). The HE analysis of the implanted NFABNS (Nano group) confirmed that these novel substitutes supported an active an abundant PNs regeneration process (Figures 3A,B). Regeneration took place through the connective tissue covering the implanted substitutes with a similar histological pattern than the observed in the auto group, meaning the presence of newly formed nerve fascicles and PNFs. In addition, like in Auto group, some degree of PN regeneration was also observed associated to the surrounding muscle tissues (Figure 3B, inset). Concerning the implanted substitutes, they were mostly biodegraded after 14 weeks. The biodegradation process was restricted to the biomaterial surface and composed by a well delimited inflammatory reaction composed by well-organized mononuclear macrophages and some perivascular white blood cells (Figures 3A,C). In the case of PNs defects repaired by collagen conduits filled with NFABNS (Coll-Nano group), histology revealed a less abundant regenerating nerve tissue than in Auto and Nano groups. In this group, the regeneration was restricted to the ECM area between the collagen conduit wall and the internal biodegradation process of the NFABNS. The regenerating tissue was composed by poorly organized newly formed fascicles. Concerning the biomaterials, the collagen conduits were well-preserved with an associated biodegradation process by giant multinuclear cells and some mononuclear infiltration. The intraluminal NFABNS showed the same biodegradation process than the one observed in Nano groups, although the structure was more preserved (Figures 3A,C). Finally, TEM analysis confirmed that the NFABNS in Nano and Coll-Nano groups was actively degraded by mononuclear macrophages (Figure 3D).

**FIGURE 4:**
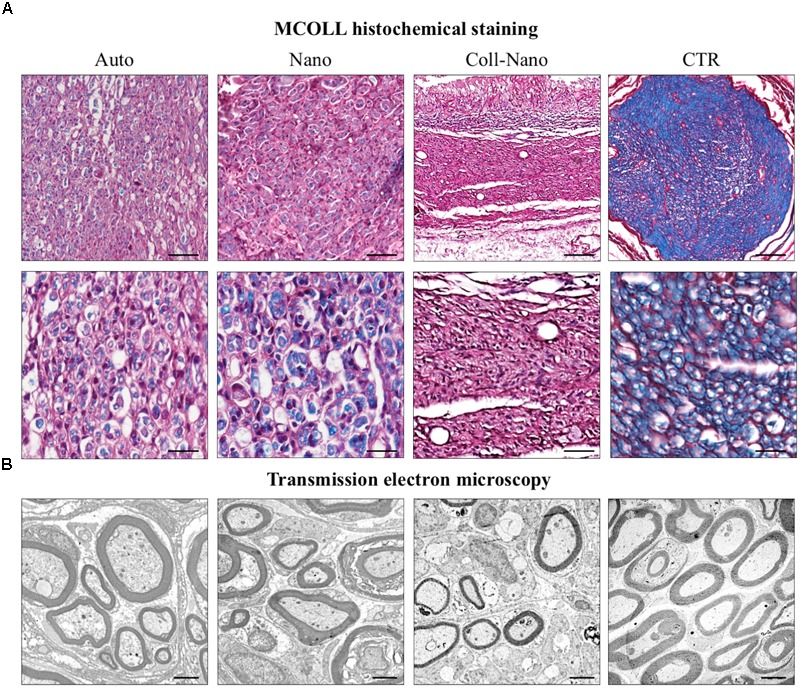
Histochemical and ultrastructural analysis of peripheral nerve regeneration and myelination. Figures in **(A)** shows the peripheral nerve regeneration pattern, degree of myelination (blue histochemical reaction) and collagen reorganization (red) with MCOLL histochemical staining at moderate and higher magnification in each experimental condition (Auto, Nano, and Coll-Nano) and native control (CTR). TEM images in **(B)** confirm the presence of myelinated peripheral nerve fibers in all experimental groups and CTR. Scale bar = 100 μm (upper images) and 50 μm (lower images) in **(A)** and 2 μm in **(B)**.

The analyses of myelin and collagen fibers content carried out with MCOLL histochemical method demonstrated that the PN regeneration process was accompanied by certain degree of myelination and collagen extracellular matrix reorganization (Figure 4A). The analysis of Auto group revealed a high amount of myelinated newly formed PNFs immersed in a loose collagen extracellular matrix (Figure 4A). Similarly, in Nano group abundant myelinated newly formed PNFs were observed in the regenerating tissue, but the ECM collagen resulted to be more abundant, especially around the newly formed nerve fascicles (Figure 4A). In the case of Coll-Nano group, evident histochemical reaction for myelin was not observed, although an important amount of collagen was detected accompanying the PN regeneration process (Figure 4A). Finally, TEM analysis confirmed the presence of newly formed PNFs with a well-formed myelin sheath as well as unmyelinated ones in both Auto and Nano groups. Interestingly, TEM analysis confirmed the presence of unmyelinated and myelinated PNFs in the Coll-Nano group, but the myelin sheath was considerably thinner and less organized than the myelin sheath observed in the other experimental groups (Figure 4B). Despite the high degree of myelination observed in Auto and Nano groups, light and electron microscopy findings were not comparable to the PNFs thickness and regularity observed in the control (Figures 4A,B).

**FIGURE 5:**
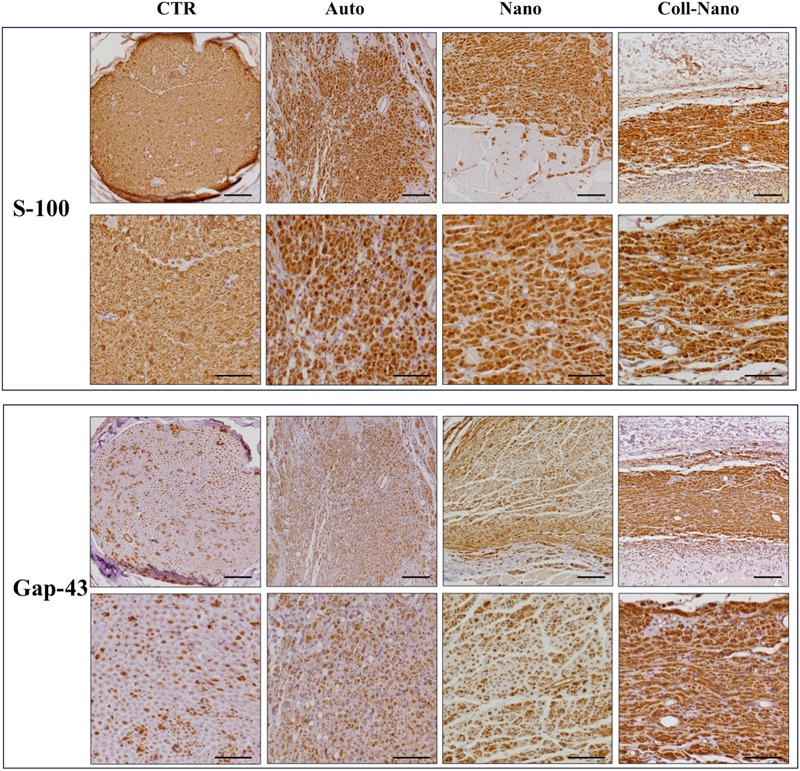
Immunohistochemical evaluation of Schwann cells and regenerating axons. The immunohistochemical staining (brown reaction) of S-100 was used to reveal the presence and distribution of Sch whereas Gap-43 was used as a marker of newly formed regenerating axons. Both immunostaining were performed in each experimental group (Auto, Nano, and Coll-Nano) and native control (CTR). Scale bar = 100 μm in lower magnifications and 50 μm higher magnification images.

In order to confirm the PN regeneration process, the SCs and newly formed axons were immunohistochemically identify by using antibodies against S-100 and GAP-43 proteins, respectively (Figure 5). The immunohistochemical analysis of Auto group revealed an abundant and consistent immunoreaction for S-100 and GAP-43 at the intrafascicular and interfascicular levels, confirming the presence of an active PN regeneration process as previously observed by the histological, histochemical and ultrastructural analyses (Figure 5). In the Nano group, an abundant immunoreaction for S-100 and GAP-43 was found associated with the regenerating tissue with a similar pattern than the one observed in the Auto group (Figure 5). The immunohistochemical study of Coll-Nano group confirmed the presence of an active PN regeneration process, but it was not comparable to the pattern and amount observed in Nano and Auto groups (Figure 5).

### Results of Muscle Morphometry and Histology

**FIGURE 6:**
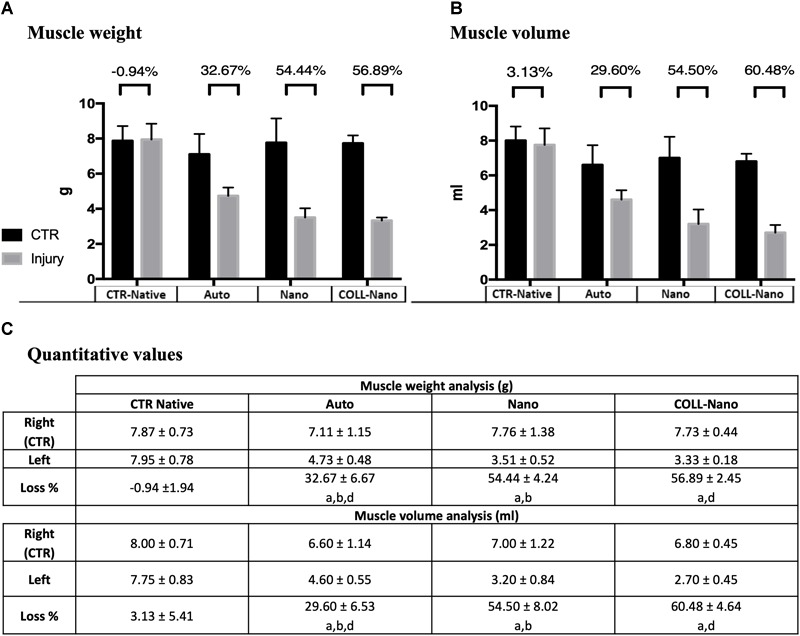
Quantitative results of muscle’s weight and volume loss. Graphics **(A,B)** show the muscle weight and volume quantitative results of the injured (gray) and right leg control (black) of the operated animals of each experimental condition (Auto, Nano, and Coll-Nano) and native control (CTR). Table in **(C)** shows the weight and volume mean ± standard deviation values in grams (g) and milliliters (ml), respectively. Furthermore, the muscle atrophy is indicated by the % of loss between the CTR and injured leg of each animal. In this study, significant differences are indicated as follows: ^a^Significant differences with CTR group. ^b^Significant differences between Auto vs. Nano group. ^c^Significant differences between Nano vs. Coll-Nano group. ^d^Significant differences between Auto vs. Coll-Nano group. *p* < 0.05 values were considered statistically significant in two-tailed tests.

Muscle atrophy is a well-known consequence that takes place after PN damage, and this process is an acceptable and informative indicator of the degree of muscle reinnervation following PN repair and regeneration (Vleggeert-Lankamp, 2007; Siemionow et al., 2011). The quantitative analysis of lower leg muscles innervated by the repaired sciatic nerves after 14 weeks revealed an important *w* and *v* loss as compared to right control legs in each group (Figure 6). In general, healthy legs weight ranged between 7.8 and 7.1 g (mean 7.5 ± 0.2 g) and the volume from 8 to 6.6 ml (mean 7.1 ± 0.5 ml), while operated legs ranged from 3.3 to 4.7 g (mean 3.8 ± 0.6 g) and 2.7 to 4.6 ml (mean 3.5 ± 0.8 ml) (Figure 6). When comparing the percentage of weight and volume loss a slight difference in in the CTR-Native group was found (0.94% and 3.1%, respectively). Concerning the operated animals, the lower percentage loss was found in Auto group, where animals loss 32.67% of the weight and 29.6% of the volume (Figure 6). When animals were repaired with bio-artificial nerve substitutes the percentages of weight and volume loss were significantly higher in Nano (54.4% of weight and 54.5% of volume) and especially in Coll-Nano (56.8% of weight and 60.4% of volume) groups as compared to animals from CTR-Native and Auto group (Figure 6). Although the percentage of loss was higher in animals from Coll-Nano group than Nano group, these differences were not statistically significant (*p* = 0.251 for weight and *p* = 0.281 for volume).

**FIGURE 7:**
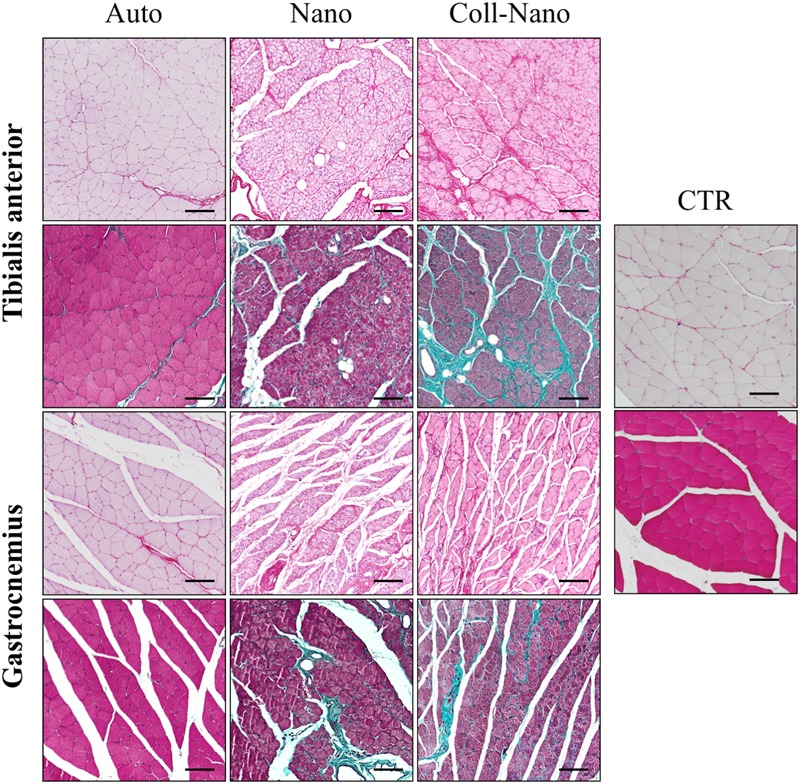
Histological profile of muscles innervated by repaired peripheral nerves. Figures show the histological pattern of the tibialis anterior and the gastrocnemius muscles stained with picrosirius (upper images for each) and Masson trichrome (lower images for each) methods of each experimental condition (Auto, Nano, and Coll-Nano) and native control (CTR). Observe that the histological pattern of Auto groups was comparable to the native control whereas different degrees of atrophy and even fibrosis can be observed in Nano and especially Coll-Nano groups. Scale bar = 100 μm in each.

The histochemical analyses of the transversal sections of the tibialis anterior and gastrocnemius muscles confirmed the EMG findings and especially the different degree of atrophy observed by the morphometric evaluation among the experimental groups (Figure 7). In Auto group, histology revealed slight signs of atrophy in both muscles analyzed. The histological pattern was characterized by the presence of randomly distributed well-delimited rhabdomyocytes with certain signs of atrophy, such as nuclear internalization and cell size reduction. In addition, we did not observe signs of fibrosis or adipose tissue infiltration with PS and MST methods (Figure 7). In general, muscles in this group showed a similar histological pattern than healthy muscles used as controls (Figure 7). In the Nano group, the degree of atrophy in both muscles was more evident than in Auto group. In this case, a grouped atrophy was observed, which was characterized by the presence of groups of small and angular rhabdomyocytes. Concerning the ECM, histochemistry revealed a slight increase of the collagen content as well as the presence of some adipocytes (Figure 7). The degree of atrophy observed in Coll-Nano group was considerably higher than in Auto and Nano groups. In this group, both muscles were mostly atrophic, composed by small muscle fascicles containing moderately to severely atrophied rhabdomyocytes. Furthermore, a considerable increase of collagen fibers and some occasional adipocytes were observed (Figure 7). Muscle histological analyses confirmed the morphometric and EMG results.

## Discussion

Here, we report an *in vivo* preclinical evaluation of two novel tissue engineering approaches for the repair of a 10-mm nerve gap in the sciatic nerve of rats: a multilayered NFABNS and the NFABNS used as intraluminal fillers of NeuraGen^®^ conduits. The PNs regeneration process and functional recovery were assessed by using clinical, functional and histological analyses.

In PNTE, bioartificial substitutes must have adequate structural, physical and biological properties to successfully repair nerve defects supporting, and ideally increasing, the regeneration process and functional recovery (Daly W. et al., 2012; Carriel et al., 2014a; Wieringa et al., 2018). From the surgical perspective, it is important that nerve substitutes may respond to specific anatomical requirements (e.g., length, diameter, number of fascicles), should be easy to handle and to suture to ensure an adequate tension-free PN repair, and should be available for use in a reasonable period of time (Carriel et al., 2014a; Gu et al., 2014; Pedrosa et al., 2017). In this regard, the current gold standard technique, the nerve autografts, effectively provides adequate biological and physical properties with an acceptable functional recovery. However, the use of sensory donor nerves to repair motor ones rarely respond to the anatomical needs (Brenner et al., 2006) and the well-known limitations of nerve autograft, and specially allografts, urge researchers to find more efficient alternatives (Daly W. et al., 2012; Carriel et al., 2014a). Although promising, most currently available PNTE strategies cannot be manufactured in an opportune range of time or, as it is the case of FDA-approved commercial devices, they are available with a pre-established range of dimensions and may hardly fulfill the anatomical demands.

In this study, we show the suitability of two natural biomaterial-based TE strategies for PN repair, which were generated following a previously described controlled and highly reproducible procedure (Carriel et al., 2017d). On the one hand, with our NFABNS, it was possible to successfully recreate the shape, diameter and length of the PNs to be repaired, thus demonstrating that the NFABNS can respond to specific anatomical needs. On the other hand, the NFABNS demonstrated to be suitable for use as intraluminal filler of NeuraGen^®^ conduits. Concerning the design of the NFABNS, they were generated by rolling thin layers of NFAH to generate consistent multilayered rods containing viable, proliferating (positive for Ki-67 and PCNA) and functional ADMSCs (Carriel et al., 2017d). In addition, this simple, fast and economic procedure has the advantage that it could be programmed some hours before the surgery, which may fulfill the time requirements for an opportune PN repair (Pedrosa et al., 2017; Zhang and Rosen, 2018). Moreover, the NFABNS has been demonstrated to be surgically easy to handle with adequate mechanical stability and flexibility, which allowed a tension-free repair just like nerve autografts. Interestingly, despite these advantages, tensile test demonstrated that our NFABNS were not fully comparable to the biomechanical response of native rat sciatic nerves (e.g., 0.30 ± 0.04 MPa vs. 8.5 ± 2.48 MPa Young’s Modulus mean values, respectively) (Carriel et al., 2017d; Philips et al., 2018a,b). Nonetheless, here we demonstrated that, independently of these biomechanical differences, our NFABNS were consistent enough to successfully repair all defects ensuring the nerve continuity after 14 weeks. The potential suitability of the use of NFABNS in PN repair is supported by previous studies, in which natural biomaterial-based substitutes with comparable multilayered 3D design were successfully used *in vivo*. In this context, plastic compressed, multilayered, and even multifasciculated, collagen-based rods containing different cell sources were previously used to bridge 8-mm (Schuh et al., 2018) and 1.5-mm defects in rat sciatic nerves (Georgiou et al., 2013, 2015). Similarly, the use of multilayered acellular small intestinal submucosa coated with SC supported tissue regeneration in a 7-mm sciatic nerve defect in rats (Hadlock et al., 2001). Unfortunately, none of these reports provided information concerning the biomechanical properties of these promising engineered substitutes, and therefore these aspects as compared to our NFABNS remain unknown.

Concerning the use of NeuraGen^®^ conduits filled with NFABNS in PN repair, this strategy allowed us to efficiently repair all nerve defects after 14 weeks without macroscopic complications. Technically, this strategy was considerably faster and easier than the use of NFABNS alone or nerve autografts because repair was done following the conventional tubulization technique. Regarding the suitability of this combined strategy in PNs repair, it is well-accepted that the use of conduits containing intraluminal fillers, especially those containing cells, is able to enrich the regenerative microenvironment with the consequent enhance of PN regeneration and functional recovery (Pedrosa et al., 2017; Gonzalez-Perez et al., 2018; Ronchi et al., 2018; Tajdaran et al., 2018; Wieringa et al., 2018). In this study, the incorporation of NFABNS into NeuraGen^®^ conduits prevented the deformation or compression of these commercial devices during the period analyzed. Deformation, compression and early reabsorption of some hollow conduits has been described associated to this technique or to the use of vein grafts in PNs reconstruction (Moore et al., 2009; Hernandez-Cortes et al., 2010; Wangensteen and Kalliainen, 2010; Daly W. et al., 2012; Liodaki et al., 2013; Papalia et al., 2013; Carriel et al., 2014a; Krarup et al., 2017). In this regard, engineered neural tissue-like substitutes based on cellular self-aligned and plastic compressed collagen hydrogels were covered by NeuraWrap^TM^ (Integra, United States) devices and successfully used to repair a critical nerve gap of 15-mm in rats (Georgiou et al., 2013). Furthermore, the use of NFABNS to fill NeuraGen^®^ conduits is especially supported by our previous studies in which these conduits filled with uncompressed FAH containing ADMSCs successfully repaired 10-mm nerve gaps in rats, supporting tissue regeneration and functional recovery after 12 weeks (Carriel et al., 2013, 2017c). Finally, all these previous studies support the potential surgical usefulness of the use of NFABNS alone or as intraluminal filler of nerve conduits in PNs repair.

Clinical and functional assessments are needed to determine the degree of functional recovery following PN repair (Vleggeert-Lankamp, 2007; Siemionow et al., 2011; Carriel et al., 2014b; Navarro, 2016). This time-course study demonstrated that the created injury severely compromised clinical and functional parameters of all animals after 4 weeks, followed by a partial recovery after 12 weeks. Clinically, all operated animals had self-amputations over the time, but they were consistently higher in the autograft group, although differences were not statistically significant. Concerning the presence of neurotrophic ulcers, which are associated to an impairment of the sensitive and motor functions (den Dunnen and Meek, 2001; Vleggeert-Lankamp, 2007; Carriel et al., 2013), they were surprisingly absent in animals treated with NeuraGen^®^ conduits filled with NFABNS over the time. However, ulcers were found in animals that received autograft (20%) and NFABNS (40%), without significant differences after 12 weeks, being these findings in agreement with previous studies (Meek et al., 1999; den Dunnen and Meek, 2001). In addition to these parameters, experimentally induced sciatic nerve injuries are associated to muscle dysfunction and neurogenic muscle retraction, which can be reflected by foot length alterations (Kim et al., 2007; Carriel et al., 2013; Kappos et al., 2015). In this study, a higher degree of neurogenic muscle retraction was observed in animals treated with autograft, while engineered strategies (NFABNS and filled conduits) were associated to better results. The development of these injuries at the foot level can be explained by a partial recovery of the sensitive and motor functions assessed by pinch and toe-spread tests. Our results were especially favorable with the use of NFABNS as compared to the autograft, being less favorable when nerves were repaired with filled conduits. However, these results were not comparable to the normal function observed in healthy animals. These results are in line with previous studies. For example, the use of fibrin conduits containing different kinds of ADMSCs demonstrated comparable functional recovery than autografts in the repair of 10-mm gaps in rats after 12 weeks (Kappos et al., 2015). In addition, a recent study demonstrated successful functional recovery through the use of chitosan conduits filled with cellular self-aligned collagen hydrogels in the repair of a critical size defect of 15-mm in rats (Gonzalez-Perez et al., 2018).

In order to accurately determine the degree of muscle denervation and reinnervation, EMG studies of gastrocnemius and tibialis anterior muscles were conducted as previously recommended (Vleggeert-Lankamp, 2007; Carriel et al., 2013, 2014b; Navarro, 2016). These analyses confirmed a high degree of muscle denervation at 4 weeks in all animals, as expected. Interestingly, after 12 weeks, a decrease of muscle denervation and an increase of muscle reinnervation were demonstrated in animals treated with NFABNS and autografts. However, certain degree of denervation was still present in the NFABNS group, especially when filled conduits were used. Interestingly, the EMG profile of animals treated with NFABNS resulted to be more favorable than the results previously obtained with the use of NeuraGen^®^ conduits filled with cellular and acellular uncompressed FAH in the same animal model and period of analysis (Carriel et al., 2013). Furthermore, our EMG results are in accordance with the percentage of *w* and *v* loss results. In fact, higher percentages of *w* and *v* loss were obtained with the use of filled conduits, followed by the use of NFABNS, being these results significantly higher than the obtained with autograft technique. These findings were later confirmed by the muscle histochemical analyses which clearly revealed slight, moderate and severe rhabdomyocytes atrophy in autograft, NFABNS and filled conduits groups, respectively. Finally, our results suggest that the use of NFABNS in PN repair promote an acceptable, and in some aspect equivalent clinical and functional recovery profile than the use autograft technique in the repair of 10-mm nerve gap in rats, being these results supported by comparable studies and engineered models (Georgiou et al., 2013, 2015; Jesuraj et al., 2014; Kappos et al., 2015; Pedrosa et al., 2017; Schuh et al., 2018; Wang and Sakiyama-Elbert, 2018).

Histological analyses are useful tools in PN regeneration research being an essential complement to the clinical, functional and electrophysiological investigation techniques (Vleggeert-Lankamp, 2007; Carriel et al., 2014b; Geuna, 2015). In this study, light microscopy and TEM histology were crucial to demonstrate an active PN regeneration process at the middle portion of all substitutes. However, differences in the amount, regenerative tissue distribution pattern and host tissue response to the implanted grafts were detected. Autograft group histology confirmed the presence of newly formed PN fascicles with moderate myelination (MCOLL and TEM results) along the connective tissue layers and especially, at the intrafascicular level. A comparable regeneration process was observed in NFABNS groups, although it was restricted to the connective tissue covering the implanted substitutes and not through the substitute layers. Surprisingly, tissue regeneration in filled conduits was considerably less abundant and poorly myelinated than the in autograft and NFABNS groups. In fact, the nerve tissue regeneration was restricted to the intraluminal area just around the intraluminal surface of the NFABNS. In summary, histology demonstrated that NFABNS – used alone or as intraluminal filler – did not promote nerve tissue regeneration through its biomaterials layers. Nevertheless, the direct use of NFABNS in PN repair resulted to be an efficient physical platform to keep both nerve stumps connected, supporting and guiding nerve tissue regeneration through its surrounding connective tissue, thus reaffirming the clinical, functional and EMG results. Therefore, our histological findings are in accordance with the active nerve tissue regeneration obtained by the use of comparable strategies (Hadlock et al., 2001; Georgiou et al., 2013, 2015; Ronchi et al., 2018; Schuh et al., 2018). In relation to the less favorable results obtained with filled NeuraGen^®^ conduits, our histological analyses suggest that this finding could be related to physical factors (Daly W. et al., 2012; Carriel et al., 2014a). It is well accepted that nerve conduits provide a close and controlled protective microenvironment that guide and support nerve tissue regeneration in non-critical nerve gaps (Daly W. et al., 2012; Carriel et al., 2013, 2014a; Krarup et al., 2017; Pedrosa et al., 2017; Ronchi et al., 2018). In this regard, we hypothesize that the combined use of an external conduit filled with a highly dense NFABNS may reduce the area needed for an optimal tissue regeneration, and this would explain the poor functional and electrophysiological recovery observed with this strategy. In fact, it was experimentally demonstrated that low-density intraluminal fillers enhance regeneration process and functional recovery (Daly W. et al., 2012; Daly W.T. et al., 2012; Lee et al., 2012; Carriel et al., 2013, 2014a, 2017c; Gu et al., 2014; Wieringa et al., 2018). On the other hand, highly dense and slowly degrading intraluminal fillers can reduce the area available for tissue regeneration, thus delaying or even inhibiting tissue regeneration (Labrador et al., 1998; Yao et al., 2010; Daly W. et al., 2012; Daly W.T. et al., 2012; Carriel et al., 2014a).

In relation to the host response to our biomaterials, histological analyses confirmed that NFABNS, used alone or as intraluminal fillers, were progressively biodegraded by a local inflammatory host response mainly consisting of macrophages. These findings are in agreement with previous studies where FAH-based substitutes were reabsorbed by a comparable biodegradation process in some weeks (Carriel et al., 2012, 2013; Fernandez-Valades-Gamez et al., 2016; Martin-Piedra et al., 2017). Besides, histology also confirmed that NeuraGen^®^ conduits protect the intraluminal NFABNS from host tissue biodegradation. This could reduce the intraluminal area needed for an optimal tissue regeneration and would reaffirm the hypothesis discussed above.

Finally, the use of cellular systems in PN repair demonstrated to be an efficient alternative to increase regeneration and functional recovery (Carriel et al., 2013, 2014a, 2017c; Kappos et al., 2015; Gonzalez-Perez et al., 2018; Zhang and Rosen, 2018). In this study, autologous undifferentiated ADMSCs were used to functionalize the NFABNS due to their well-demonstrated positive impact on PN repair (Kalbermatten et al., 2008; Lopatina et al., 2011; Gu et al., 2014; Faroni et al., 2016; Carriel et al., 2017b). In this study, acceptable PN regeneration and functional recovery profiles were obtained with the direct use of NFABNS in PN repair. However, due to technical reasons, it was not possible to identify the cells implanted within the NFABNS. Therefore, we cannot directly attribute to the cells the positive effect on PN regeneration obtained with our NFABNS. Therefore, the fate and potential role of these cells following *in vivo* implantation remains unknown and should be determined in future studies. In this regard, previous studies suggested that ADMSCs could contribute to the regeneration process through their differentiation to a SCs-like phenotype, releasing essential neurotrophic factors and collaborating with the synthesis of an essential ECM (Salgado et al., 2010; Tomita et al., 2012; Carriel et al., 2013; Kappos et al., 2015; Faroni et al., 2016; Zhang and Rosen, 2018).

## Conclusion

In conclusion, the present study suggests that NFABNS support a closely comparable functional recovery and tissue regeneration than autograft technique. Overall results suggest that NFABNS may have potential clinical usefulness in future clinical trials in humans. However, further research should confirm their positive impact on the reconstruction of critical nerve defects and should improve the properties of these bioartificial tissue substitutes.

## Author Contributions

VC, MA, and AC designed the experiments. VC, JC-A, and FC wrote the article. VC, JC-A, FC, OR, and DD-H performed the laboratory analyses. VC, JC-A, EM, JS-M, SG-G, and OR performed the functional and clinical analyses. VC, JC-A, FC, MA, and EM analyzed the results.

## Conflict of Interest Statement

The authors declare that the research was conducted in the absence of any commercial or financial relationships that could be construed as a potential conflict of interest. The handling Editor declared a past co-authorship with several of the authors VC, AC, and MA.
